# 2-[(3-Aminoalkyl-(alkaryl-,aryl-))-^1^H-1,2,4-triazol-5-yl]anilines: synthesis and anticonvulsant activity

**DOI:** 10.3906/kim-2002-24

**Published:** 2020-06-01

**Authors:** Yulya MARTYNENKO, Galina BEREST, Nina BUKHTIAYROVA, Igor BELENICHEV, Oleksiy VOSKOBOINIK, Sergiy KOVALENKO

**Affiliations:** 1 Department of Organic and Bioorganic Chemistry, Pharmaceutical Faculty, Zaporizhzhia State Medical University, Zaporizhzhia Ukraine; 2 Department of Pharmacology and Medical Formulation, Pharmaceutical Faculty, Zaporizhzhia State Medical University, Zaporizhzhia Ukraine

**Keywords:** N -acylated{([1,2,4]triazolo[1,5-c]quinazolin-2-yl)alkyl-(alkaryl-, aryl-)}amines, 2-[(3-aminoalkyl-(alkaryl-,aryl-))-1H-1,2,4-triazolo]anilines, hydrazinolysis, acidic hydrolysis, anticonvulsant activity

## Abstract

The presented work is devoted to the development of synthesis methods for novel 2-[(3-aminoalkyl-(alkaryl-, aryl-))-1H-1,2,4-triazolo]anilines. Abovementioned compounds were obtained via hydrazinolysis (Ing-Manske procedure) and acid hydrolysis of corresponding N -acylated{([1,2,4]triazolo[1,5-c]quinazolin-2-yl)alkyl-(alkaryl-, aryl-)}amines. The regioselectivity of hydrazinolysis and hydrolysis were established. The features of spectral characteristics werestudied and discussed. Characteristic patterns of protons signals splitting in ^1^H NMR of the synthesized compounds were established. The effect of the synthesized compounds on the pentylenetetrazol seizures was studied. It was found that according to some indicators, anticonvulsant activity of 2-[(3-aminoalkyl-(alkaryl-, aryl-))-1H-1,2,4-triazolo]anilines superior or comparable with effect of the reference drug “Lamotrigine”. It is a valid argument for their further structural modification, in-depth study of activity mechanisms and further study of anticonvulsant activity on other experimental seizures models.

## 1. Introduction

The number of patients with epilepsy and seizures has increased significantly in recent years. The abovementioned conditions are the result of organic or functional damage in brain areas and may be caused by various factors such as injuries, circulatory disorders, somatic or infection diseases, brain tumours and abnormalities, metabolic disorders, etc. Oxidative stress is activated in the epileptic foci in case of local tissue hypoxia. It leads to overproduction of active oxygen forms (AOF) by the neurochemical (glutamate-, aspartate) neuron systems [1]. The AOF accumulation and activation of free radical oxidation processes lead to the oxidative modification of lipid and protein moieties in membranes. The abovementioned processes result the changes in GABA-A receptors sensitivity, the damage of excitatory and inhibitory neurotransmitters receptors, the synthesis violation and inappropriate releasing of neurotransmitters into the synaptic cleft and the impaired generation and conduction of nerve impulse [2]. Among the antiepileptic drugs used for the correction of listed above states are: glutamate releasing inhibitors (phenytoin, lamotrigine), GABA-A receptor (benzodiazepine), and GABA transaminase inhibitors (vigabatrin), NMDA-receptor antagonists (valproic acid), GABA reuptake inhibitor from the synaptic cleft (tiagabine), blockers of T-type calcium channels (ethosuximide) [3,4]. Recently it has been established, that H3R receptors play an important role in the pathogenesis of convulsive disorders. They control the synthesis and releasing of histamine and effect on the releasing of other neurotransmitters in variable areas of the brain [5]. Recent achievements in elaboration of antagonists/agonists of H3R receptors revealed the new direction for searching drugs, capable to treat neuropsychiatric disorders [5]. Nowadays most of them (thioperamide, cipralisant, ciproxifan, pitolisant, etc) are at different stages of clinical implementation for treatment of various disorders (narcolepsy, depression, schizophrenia, epilepsy, etc.). Despite the fact that the number of H3R receptors antagonists/agonists is steadily increasing, almost all of them have a similar structure: the main moiety (secondary or tertiary amine), connected *via* “linker” group (alkyl group) with the central nucleus (heterocycle or heteroatom) is replaced by various structural elements with certain physicochemical properties (Figure) [6]. Considering the abovementioned, we have made an attempt to synthesize the similar compounds, containing alkyl-, alkaryl- and arylamine groups in their structure, combined with triazolo[c]quinazoline (1) or triazole moieties (2) and to study their effect on pentylenetetrazol convulsions (Figure). Moreover, compounds with anticonvulsant activity were identified among mentioned heterocycles. Some of them are characterized by affinity to specific receptors [7–12].

**Figure:**
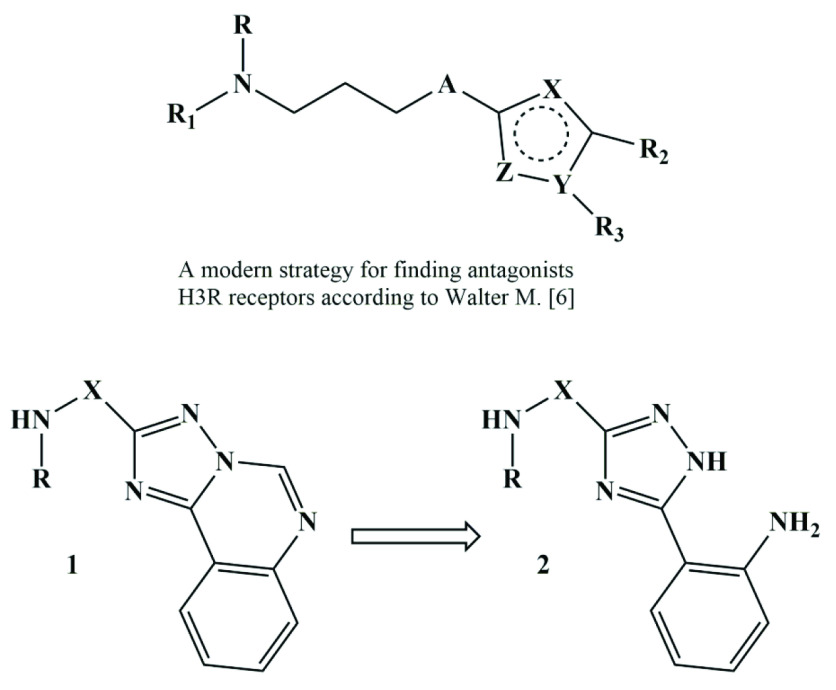
The strategies for search of H3R receptor antagonists/agonists as promising agents for neuropsychiatric disorders treatment.

Therefore, the aim of the present work was to develop methods for the synthesis of unknown 2-[(3- aminoalkyl-(alkaryl-, aryl-))-1 *H* -1,2,4-triazolo]anilines and to study spectral characteristics and anticonvulsant activity.

## 2. Materials and methods

### 2.1. Materials

Melting points were determined in open capillary tubes in a “Stuart SMP30” apparatus and were uncorrected. The elemental analyses (C, H, and N) were performed using the “ELEMENTAR vario EL cube” analyser. ^1^H NMR spectra (400 MHz) were recorded on a “Varian-Mercury 400” spectrometer with SiMe4 as internal standard in DMSO-d6 solution. LC/MS spectra were recorded using chromatography/mass spectrometric system, which consists of high-performed liquid chromatograph “Agilent 1100 Series” equipped with diodematrix and mass-selective detector “Agilent LC/MSD SL” (atmospheric pressure chemical ionization–APCI). Ionization mode was a concurrent scanning of positive and negative ions in the mass range of 80–1000 m/z.

### 2.2. General method

N -acylated{([1,2,4]triazolo[1,5-c]quinazolin-2-yl)alkyl-(alkaryl-, aryl-)}amines (1.1-1.3, 2.1-2.5, 4.1-4.3) were synthesized according to the known methods [13,14]. Synthetic procedures were conducted according to common approaches for potential biologically active substances search. Reagents were supplied by “Sigma-Aldrich” (Missouri, USA) and “Enamine Ltd” (Kiev, Ukraine).

#### 2.2.1. The general method for the synthesis of 2-(3-(aminoalkyl- (aralkyl-, aryl-)-1H-1,2,4-triazolo-5-yl)anilines (3.1-3.3)

**Method A.** To 0.005 M of the corresponding N -acylated derivatives (1.1-1.3), (2.1-2.3), (4.1-4.3) of {[1,2,4]triazolo[1,5-c]quinazolin-2-yl)methyl-(phenethyl-, phenyl-)}amines in 10 mL of methanol 2.5 mL (0.05 M) of hydrazine hydrate was added and refluxed until complete dissolution (20–40 min). The solvent and hydrazine were evaporated under vacuum, cold water was added, and the mixture was triturated. Hydrochloric acid was added to pH 5-6, the resulting precipitate was filtered. If it was necessary, the precipitate could be crystallized from methanol.

**Method B.** To 0.005 M of the corresponding N -([1,2,4]triazolo[1,5-c]quinazolin-2-ylmethyl)acetamides (1.1-1.3) 10 mL of 10% hydrochloric acid was added and refluxed during 2 h. The solvent was evaporated under vacuum, cold water was added and the mixture was acidified to pH 6. The resulting precipitate was filtered. If it was necessary, the precipitate could be crystallized from methanol.

#### 2.2.2. 2-(3-(Aminomethyl)-1H-1,2,4-triazol-5-yl)aniline (3.1)

Yield: 37.0%; M.p. 268–270 °C; ^1^H NMR δ (ppm), J (Hz): 14.39 (br.s., 1H, N H -triazol), 8.85, (b.s., 2H, CH_2_N H_2_), 7.79 (d, 1H, 2-NH_2_C_6_H_4_- H-3), 7.07 (t, J = 7.3, 1H, 2-NH_2_C_6_H_4_- H-5), 6.79 (d, J = 8.2, 1H, 2-NH_2_C_6_H_4_- H-6), 6.56 (t, J = 7.4, 1H, 2-N H_2_C_6_H_4_- H-4), 4.13 (br.s., 2H, -C H_2_NH_2_) ; LC-MS, m/z = 190 [M+l]; Anal. Calcd. for C_9_H_11_N_5_: C, 57.13; H, 5.86; N, 37.01; Found: C, 57.16; H, 5.84; N, 37.00.

#### 2.2.3. 2-(3-(4-(Aminomethyl)phenyl)-1H-1,2,4-triazol-5-yl)aniline (3.2)

Yield: 51.3%; M.p. 157–159 °C; ^1^H NMR δ (ppm), J (Hz): 8.03 (d, J = 8.1, 2H, -C_6_H_4_CH_2_NH_2_H-2,6), 7.85 (d, J = 7.2, 1H, 2-NH_2_C_6_H_4_- H-3), 7.42 (d, J = 8.0, 2H, -C_6_H_4_CH_2_NH_2_H-3,5), 7.07 (t, J = 7.6 Hz, 1H, 2-NH_2_C_6_H_4_ - H-5), 6.78 (d, J = 8.2, 1H, 2-NH_2_C_6_H_4_ - H-6), 6.59 (t, J = 7.4, 2-NH_2_C_6_H_4_ - H-4), 6.51 (br.s., 2H, 2-NH_2_C_6_H_4_ -), 4.97–4.40 (br.s., 2H, -C6 H4 CH_2_ NH 2) , 3.83 (s, 2H, -C_6_H_4_CH_2_NH_2_); Anal. Calcd. for C_15_H_15_N_5_: C, 67.90; H, 5.70; N, 26.40; Found: C, 67.94; H, 5.64; N, 26.42.

#### 2.2.4. 2-(3-(4-Aminophenyl)-1H-1,2,4-triazol-5-yl)aniline (3.3)

Yield: 57.9%; M.p. 290–292 °C; ^1^H NMR δ (ppm), J (Hz): 13.74 (br.s., 1H, NH-triazol), 8.01 (d, J = 6.6, 1H, 2-NH_2_C_6_H_4_ - H-3), 7.74 (d, J = 8.2, 2H, 4-NH_2_C_6_H_4_ - H-2,6), 7.02 (t, 1H, 2-NH_2_C_6_H_4_ - H-5), 6.79-6.61 (m, 3H, 2-NH_2_C_6_H_4_ - H-6, 4-NH_2_C_6_H_4_ - H-3,5), 6.57 (t, J = 7.2, 1H, 2-NH_2_C_6_H_4_ - H-4), 6.25 (s, 2H, 2-NH_2_C_6_H_4_ -), 5.24 (s, 2H, 4-NH_2_C_6_H_4_ -); LC-MS, m/z = 252 [M+l]; Anal. Calcd. for C_14_H_13_N_5_: C, 66.92; H, 5.21; N, 27.87; Found: C, 66.90; H, 5.19; N, 27.91.

Synthesized compounds 3.1-3.3 are light yellow crystalline substances soluble in DMF, DMSO, dioxane, alcohols, and insoluble in water.

### 2.3. The method for the synthesis of (5-(2-aminophenyl)-1H-1,2,4-triazol-3-yl)methaneaminium 4-oxo-3,4-dihydrophthalazine-1-olate (5.1)

To 1.64 g (0.005 M) of 2-([1,2,4]triazolo[1,5-c]quinazolin-2-yl-methyl)-1H-isoindole-1,3(2H) -dione (4.1) in 20 mL of methanol 2.5 mL (0.05 M) of hydrazine hydrate was added and refluxed during 20 min. The solvent and hydrazine were evaporated under vacuum, cold water was added, and the mixture was triturated. The resulting precipitate was filtered.

#### 2.3.1. (5-(2-Àminophenyl)-1H-1,2,4-triazol-3-yl)methanaminium 4-oxo-3,4-dihydrophthalazine-1-olate (5.1)

Yield: 86.3%; M.p. 248–250 °C; ^1^H NMR δ (ppm), J (Hz): 8.08 (dd, J = 5.6, 3.1, 2H, phthalazine H-5,8), 7.86-7.68 (m, 3H, H-3, phthalazine H-6,7), 7.03 (t, J = 7.8, 1H, H-5), 6.72 (d, J = 7.9, 1H, H-6), 6.54 (t, J = 7.4, 1H, H-4), 6.35 (s, 2H, NH_2_), 3.87 (s, 2H, CH_2_); LC-MS, m/z = 190 [M+1] (0.284 min) and 161 [M+1] (0.656 min); Anal. Calcd. for C_17_H_17_N_7_O_2_: C, 58.11; H, 4.88; N, 27.90; Found: C, 58.18; H, 4.93; N, 27.97.

### 2.4. The general method for the synthesis of N-(5-(2-aminophenyl)-1H-1,2,4-triazol-3-yl)-alkyl- (aralkyl-, aryl-)benzamides (6.1-6.3)

To 0.005 M of the corresponding N -([1,2,4]triazolo[1,5-c]quinazolin-2-yl-methyl)benzamides (2.2, 2.4, 2.5) 10 mL of a 10% hydrochloric acid was added. The formed mixture was refluxed until complete dissolution. Then solution was neutralized to pH 6. The resulting precipitate was filtered. The precipitate could be crystallized from methanol.

#### 2.4.1. N-(4-(5-(2-Àminophenyl)-1H-1,2,4-triazolo-3-yl)benzyl)benzamide (6.1)

Yield: 68.0%; M.p. 197–199 °C; ^1^H NMR δ (ppm), J (Hz): 8.92 (br.s, 1H, -NHBz), 8.12 (d, J = 8.1, 1H, NH_2_C_6_H_4_ - H-6), 7.95-7.79 (m, 5H, NH_2_C_6_H_4_ - H-4, Bz H-2,6; -CH_2_C_6_H_4_- H-3,5), 7.62–7.07 (m, 7H, NH_2_C_6_H_4_ -, H-3,5, Bz H-3,4,5, -CH_2_C_6_H_4_- H-2,6, NH_2_), 4.55 (d, J = 5.9, 2H, -CH_2_C_6_H_4_-); LC-MS, m/z = 370 [M+l]; Anal. Calcd. for C_22_H_19_Nsub>5O: C, 71.53; H, 5.18; N, 18.96; Found: C, 71.50; H, 5.22; N, 19.01.

#### 2.4.2. N-(1-(5-(2-Aminophenyl)-1H-1,2,4-triazolo-3-yl)-3-methylbuthyl)benzamide (6.2)

Yield: 55.9%; M.p. 177–180 °C; ^1^H NMR δ (ppm), J (Hz): 13.70 (br.s., 1H, NH-triazol), 8.60 (br. s, 1H, -NHBz), 7.92 (d, J = 7.2 Hz, 3H, H_2_NC_6_H_4_- H-6, Bz H-2,6), 7.67–7.17 (m, 3H, Bz H-3,4,5), 7.03 (t, J = 7.2, 1H, H_2_NC_6_H_4_- H-5), 6.72 (d, J = 8.^1^Hz, 1H, H_2_NC_6_H_4_- H-3), 6.54 (t, J = 7.4 Hz, 1H, H_2_NC_6_H_4_- H-4), 6.29 (s, 2H, NH_2_C_6_H_4_) , 5.37 (q, J = 8.6, 8.1, 1H, -CHCH_2_CH(CH_3_)_2_), 2.11-1.82 (m, 2H, -CHCH_2_ CH(CH_3_)_2_) , 1.74 (dt, J = 13.4, 6.3, 1H, -CHCH_2_ CH(CH_3_)_2_), 1.01 (dd, J = 6.2, 4.2, 6H, -CHCH_2_ CH(CH_3_)_2_); LC-MS, m/z = 350 [M+l]; Anal. Calcd. for C_20_H_23_N_5_O: C, 68.74; H, 6.63; N, 20.04; Found: C, 68.70; H, 6.71; N, 20.11.

#### 2.4.3. N-(1-(5-(2-Aminophenyl)-1H-1,2,4-triazolo-3-yl)-2-phenylethyl)benzamide (6.3)

Yield: 39.2%; M.p. 214–216 °C; ^1^H NMR δ (ppm), J (Hz): 13.74 (br.s., 1H, NH-triazol), 8.72 (br. s, 1H, -NHBz), 7.95–7.71 (m, 3H, H_2_NC_6_H_4_- H-6, Bz H-2,6), 7.54–7.43 (m, 1H, Bz H-4), 7.40 (t, J = 7.3, 2H, Bz H-3,5), 7.31 (d, J = 7.3, 2H, -CHCH_2_ C6H5 H-2,6), 7.23 (t, J = 7.4, 2H, -CHCH_2_ C6H5 H-3,5), 7.14 (t, J = 7.2, 1H, -CHCH_2_ C6H5 H-4), 7.05 (t, J = 7.3, 1H, H_2_NC_6_H_4_- H-5), 6.74 (d, J = 7.9, 1H, H_2_NC_6_H_4_- H-3), 6.56 (t, J = 7.4, 1H, H_2_NC_6_H_4_- H-4), 6.42 (s, 2H, NH_2_C_6_H_4_) , 5.51 (q, J = 8.3, 1H, -CHCH_2_ Ph), 3.63–3.16 (m, 2H, -CHCH_2_ Ph); LC-MS, m/z = 384 [M+l]; Anal. Calcd. for C_23_H_21_N_5_O: C, 72.04; H, 5.52; N, 18.26; Found: C, 71.99; H, 5.60; N, 18.32.

The synthesized compounds 6.1-6.3 are light yellow crystalline substances, soluble in DMF, DMSO, slightly soluble in dioxane, alcohols, and insoluble in water.

### 2.5. Anticonvulsant activity

Estimation of synthesized substances anticonvulsant activity was carried out on 90 white rats, the weigh 110–130 g, obtained from the nursery of the Institute of Pharmacology and Toxicology of the Academy of Medical Sciences of Ukraine (Kyiv). The study was conducted under the “Guidelines for the care and use of laboratory animals”, published in the United States by the National Institute of Health [15]. Seizures were modelled by a single subcutaneous administration of pentylenetetrazol (corazole) (Nizhpharm, Russian Federation) at a dose of 80 mg/kg [16]. One hour prior to the administration of the convulsant, the test compounds were administered intragastrically at a dose of 10 mg/kg as an aqueous suspension stabilized with Tween-80. “Lamotrigine” (PharmaStart, Ukraine) was used as a reference drug, administered similarly at a dose of 50 mg/kg. The control group of animals intragastrically received a similar volume of water with Tween-80. The determination of the testing time was based on data on the peak of anticonvulsant activity of the test compounds. The severity of the anticonvulsant effect was evaluated by the duration of the latent period of seizures, the type and duration of seizures in minutes and the mortality index.

### 2.6. Statistical analysis

Statistical data processing was performed using the “STATISTICA for Windows 6.0” (StatSoftInc., ¹AXXR712D 833214FAN5), “SPSS 16.0” (SPSS Inc, Chicago, IL, USA) and “Microsoft Office Excel 2003” software. The results are presented as mean ±standard error of the mean. Arithmetic mean and standard error of the mean were calculated for each of the studied parameters. During verification of statistical hypothesis, null hypothesis was declined if statistical criterion was P < 0.05 [17].

## 3. Results and discussion

At the first stage the removal of acyl fragment from the molecules of N -acylated {([1,2,4]triazolo[1,5-c]quinazolin-2-yl)alkyl-(alkaryl-, aryl-)}amines (1, 2, 4) was conducted by the Ing-Manske procedure [13,14]. It was found that conversion under the Ing-Manske reaction conditions for acetyl derivatives 1.1-1.3 led to the removal of the acyl group as well as to the nucleophilic cleavage of the pyrimidine ring (Scheme) [18,19].

**Scheme:**
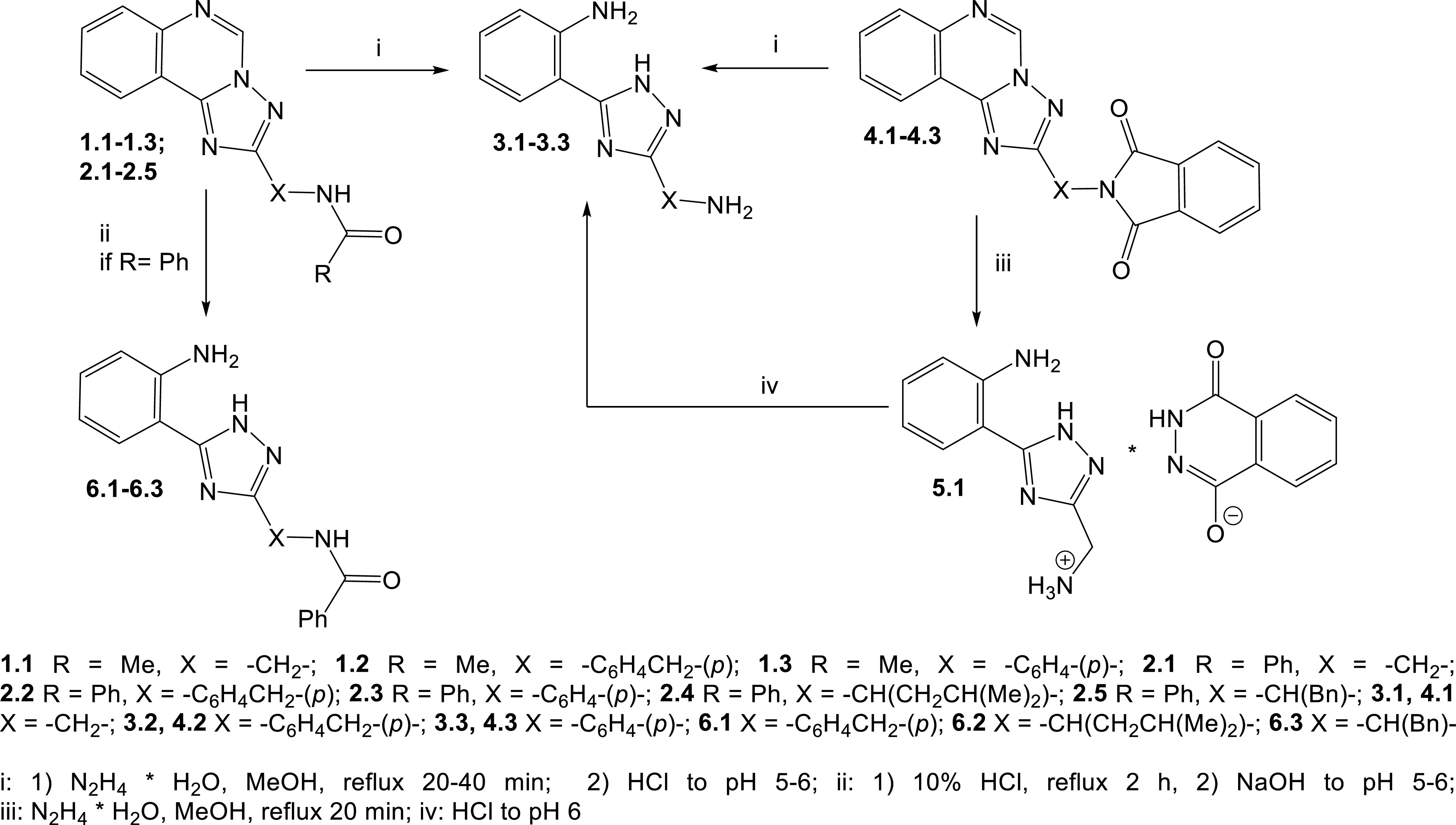
The N -acylated {([1,2,4]triazolo[1,5-c]quinazolin-2-yl)alkyl-(alkaryl-, aryl-)}amines deprotection.

Similar changes were typical for benzoyl derivatives 2.1-2.3 in the conditions of this reaction. As a result, 2-(3-(aminoalkyl-(aralkyl-, aryl-)-1H-1,2,4-triazol-5-yl)anilines (3.1-3.3) were formed with satisfactory yields. The characteristic protons signals of 2 NH_2_ groups and NH- group of the triazole cycle in the ^1^H NMR spectra indicate target compounds 3.1-3.3 formation. Thus, the signals associated with the NH_2_ group of the o-aniline moiety were observed as a broad singlet at the 6.51–6.25 ppm (3.2, 3.3) or were absent (3.1) due to solvent exchange [20]. The absence (3.3) or the broadening (3.1, 3.2) of the proton signal of the endocyclic NH-group of triazole at the 14.39 and 13.74 ppm were caused by tautomeric transformations as well. Whereas the signal that were associated with NH_2_ -group at “linker” fragment appeared as broad singlet at the 4.97–4.40 ppm (3.2) or singlet at the 5.24 ppm (3.3). The aromatic proton signals of o-aniline moiety of compounds 3.1-3.3 were recorded in a higher field compared to compounds 1 spectra, with corresponding multiplicity and chemical shift: doublet of H-3 at the 8.01–7.79 ppm, triplet of H-4 at the 6.59–6.57 ppm, triplet of H-5 at the 7.07–7.02 ppm, doublet of H-6 at the 6.79–6.78 ppm.

It is important that compounds with phthalimide fragment under the Ing-Manske reaction conditions will be subjected to cleavage (4.1-4.3, Scheme). It is obvious that the corresponding [2-(3-R-1,2,4-triazolo-5-yl)phenyl]amines (3.1-3.3) would be the result of the nucleophilic degradation of both isoindole moiety and pyrimidine cycles. Whereas primary amine with [1,2,4]triazolo[1,5-c]quinazolin fragment is expected product in case of selective isoindole cycle opening. However, the results show that compound 4.1 under the action of hydrazine hydrate yielded product 5.1. The LC-MS spectra confirmed that (5-(2-aminophenyl)-1H-1,2,4-triazolo-3-yl)methanaminium 4-oxo-3,4-dihydrophthalazine-1-olate (5.1) is the product of the reaction. It is the salt formed by the interaction of amine (3.1) with 2,3-dihydrophthalazine-1,4-dione (pKa1 = 5.87; pKa2 = 14.75) [21]. The abovementioned was additionally confirmed by the ^1^H NMR spectral characteristics of the compound 5.1. Thus, signals of cationic and anionic fragments of the molecule were present in the ^1^H NMR spectrum of salt 5.1. Thus, (5-(2-aminophenyl)-1H-1,2,4-triazolo-3-yl)methanaminium cation was characterized by aromatic protons signals of the aniline moiety with the corresponding multiplicity and chemical shift: triplet of H-4 at the 6.54 ppm, triplet of H-5 at the 7.03 ppm, doublet of H-6 at the 6.72 ppm and the doublet of H-3, which overlaps on the phthalazine cycle protons H-6 and H-7 and was observed as multiplet at the 7.86–7.68, 7.86 ppm in the spectrum. ^1^H NMR-spectrum of compound 5.1 was characterized by the signals of -NH_2_ and -CH_2_ - protons that were observed at the 6.72 ppm and 3.87 ppm, correspondingly. It clearly proves the nucleophilic cleavage of compound 4.1. In addition, the spectrum contains characteristic signals of H-5 and H-8 phthalazine cycle protons, which were observed as doublet of doublets at the 8.08 ppm (SSCC; 5.6, 3.^1^Hz), as well as the H-6 and H-7, which were highlighted above. It is important that the modification of target compound isolation procedure, namely the pH variation after the reaction and further extraction allowed to obtain compound 3.1. The established optimal synthesis conditions were implemented to the reactions for compounds 4.1-4.3. Such the corresponding 2-(triazolyl-)anilines 3.1-3.3 were also isolated (Scheme).

The possibility of compounds 1, 2, and 4 deprotection by acidic hydrolysis was studied as well. It was conducted for further development of approaches for the synthesis of original azolylanilines. It was shown that compounds 4 with phthalimide moiety cannot be hydrolysed in abovementioned conditions due to their insolubility in aqueous and aqueous alcoholic acids solutions. Whereas, N -acetyl derivatives (1.1-1.3) under acidic hydrolysis underwent hydrolytic cleavage of both the pyrimidine cycle and the acetamide moiety what yielded compounds 3.1-3.3 (Scheme). At the same time, the selective cleavage of the pyrimidine moiety in molecules of N -benzoyl-([1,2,4]triazolo[1,5-c]quinazolin-2-yl-)alkyl-(aralkyl-, aryl-)amines (2.2, 2.4, 2.5) was observed as result of acidic hydrolysis (6.1-6.3). This fact could be explained by the higher stability of the conjugated benzamide moiety.

The ^1^H NMR spectra of compounds 6.2 and 6.3 were characterized by a broad singlet signals of the protons of endocyclic NH- group in the triazole cycle at the 13.74–13.70 ppm. However, compounds 6.1-6.3 have a characteristic proton signal of benzamide group as a broad singlet at the 8.92–8.60 ppm, unlike compounds 3.1-3.3. The signals of the NH 2 group protons of the o-aniline moiety were recorded as a broad singlet at the 6.42–6.29 ppm. The signals of the o-aniline moiety protons were observed as doublets H-3 at the 6.74–6.72 ppm, triplet H-4 at the 6.56–6.54 ppm and H-5 at the 7.05–7.03 ppm. While the H-6 signal was registered together with the proton signals of the H-2,6 benzoyl group at the 7.95–7.71 ppm. The protons of other protons of this group were observed as doublet of a triplet at the 7.43 ppm (6.2, SSCC 14.6, 7.0 Hz, Bz H-3,4,5), the unsplitted triplet of H-4 and the triplet of H-3,5 at the 7.54–7.43 and 7.40 ppm (6.3, SSCC 7.3 Hz), respectively. The chemical shifts and protons multiplicity of the alkyl- (3.1, 6.2), aralkyl- (3.2, 6.1, 6.3), and aryl- (3.3) groups depend on the nature of the “linker” and correspond to the proposed structure [22].

It was established (Table) that administration of pentylenetetrazol (corazole) led to the development of epileptic seizures with the expressed tonic-clonic phase and subsequent 100% mortality of animals. Thus, in the control group, the latent period was on average 6.78 min and the duration of tonic-clonic seizures was 8.12 min. Seizures, that were observed in this group of animals had the expressive tonic-clonic character and periodically repeated. The expressive phase of tonic extension was presented as well.

**Table:** 

Compound	Latent seizure period, min	Duration of tonic-clonic seizure, min	Mortality, %
Control	6.78 ± 0.44	8.12 ± 0.64	100
1.1	15.20 ± 1.20*	5.90 ± 1.20	60*
1.2	12.10 ± 1.0*	6.88 ± 1.0	90*
1.3	19.70 ± 1.40*	7.33 ± 1.20	60*
2.2	17.10 ± 1.20*	5.75 ± 0.42	80*
2.3	22.70 ± 1.10*	5.87 ± 2.80	60*
3.1	39.10 ± 3.70*	4.77 ± 0.42*	50*
3.2	45.20 ± 3.20*	3.55 ± 0.22*	40*
3.3	47.80 ± 2.0*	3.70 ± 0.40*	40*
6.1	14.10 ± 1.0*	7.56 ± 1.12	80*
Lamotrigine	31.20 ± 1.70*	2.77 ± 0.67*	20*

Note: *significantly (P ≤ 0.05) relative to the control group of rats.

Administration of N -acetyl-([1,2,4]triazolo[1,5-c]quinazolin-2-yl-)alkyl-(alkaryl-, aryl-)amines (1.1-1.3) to experimental animals led to increase of seizure latent period up to 11.1–12.9 min, compared to control. These compounds also reduced the duration of tonic-clonic seizures up to 0.8–7.2 min and prevented animal mortality on 10–40%. It is important that in case of compounds 1.1-1.3 variation of “linker” group nature does not significantly affect on their activity. Replacing of N -acetyl (1.2, 1.3) by the N -benzoyl moiety (2.2, 2.3) without changing of the “linker” group nature allowed to identify compound 2.3 as promising anticonvulsant agents. Thus, compound 2.3 increases the latent period of seizures up to 15.9 min, reduces clonic-tonic convulsions duration up to 2.25 min and prevents animal mortality up to 60%.

Further structure modification of compounds 1 and 2, namely the removal of acyl protection, leads to increased activity. Thus, compound 3.1 with the methyl “linker” group increased the latent period more than 5.7 times compared to “Lamotrigine”. However, compound 3.1 is inferior to “Lamotrigine” by reducing the duration of tonic-clonic seizure and mortality. Replacement the methyl group (3.1) by benzyl (3.2) and phenylene (3.3) in the corresponding 2-(3-R-1H-1,2,4-triazol-5-yl)anilines led to increasing of anticonvulsant activity (Table). Thus, compounds 3.2 and 3.3 significantly reduced the seizures latent period (up to 41 min), reduced the duration of tonic-clonic seizures up to 4.4–4.6 min and prevented the mortality of animals (40%) compared to control. It is interesting that N -(4-(5-(2-aminophenyl)-1H-1,2,4-triazol-3-yl)benzyl)benzamide (6.1) is inefficient compound and significantly inferior in effect to compound 3.2. That is blocking of the benzylamino group of the compound (3.2) that lead to the loss of anticonvulsant activity. Thus, compounds 3.1-3.3 are a promising class of anticonvulsant agents, which exceed or compete with the reference drug “Lamotrigine” according to some indicators. Thus, anticonvulsant activity was found among unknown 2-(3-R-1H-1,2,4-triazol-5-yl)anilines (3.1-3.3) for the first time and it is a strong argument of their further structural modification and in-depth mechanisms of action study and research on other experimental models.

## 4. Conclusion

A system study was carried out to remove the protective group from N -acylated {([1,2,4]triazolo[1,5-c]quinazole-2-yl)alkyl(alkaryl-, aryl-)}amines by hydrazinolysis and acidic hydrolysis. Features and directions of the reaction were established. It was shown that unknown 2-[(3-aminoalkyl-(alkaryl-, aryl-))-1H-1,2,4-triazol-5-yl]anilines are a promising class of anticonvulsant agents, which exceed or compete with the reference drug “Lamotrigine” according to some indicators.
